# Supplementary material for: Direct Visualization of Temperature‐Induced Phase Separation of Completely Miscible Au─Pd Alloy by In Situ TEM
Source: Small. 2025 Jan 26;21(19):2408109. doi: 10.1002/smll.202408109 (PMC12067158; doi:10.1002/smll.202408109)
Supplement: Supplementary file 1 — Supporting Information [file SMLL-21-2408109-s006.docx]

Supporting Information

**Direct Visualization of Temperature-Induced Phase Separation of Completely Miscible Au-Pd Alloy by In-Situ TEM**

*Abhijit Roy^*^, Simon Hettler, and Raul Arenal^*^*

A. Roy, S. Hettler, R. Arenal

Laboratorio de Microscopías Avanzadas (LMA), Universidad de Zaragoza, Zaragoza, SPAIN
E-mail: [arenal@unizar.es](mailto:arenal@unizar.es), [aroy@unizar.es](mailto:aroy@unizar.es)

A. Roy, S. Hettler, R. Arenal
Instituto de Nanociencia y Materiales de Aragón (INMA), CSIC-Universidad de Zaragoza, Zaragoza 50009, Spain

R. Arenal

ARAID Foundation, Zaragoza 50018, Spain

Keywords: Au-Pd alloy, Phase separation, In-situ heating TEM, aberration-corrected TEM


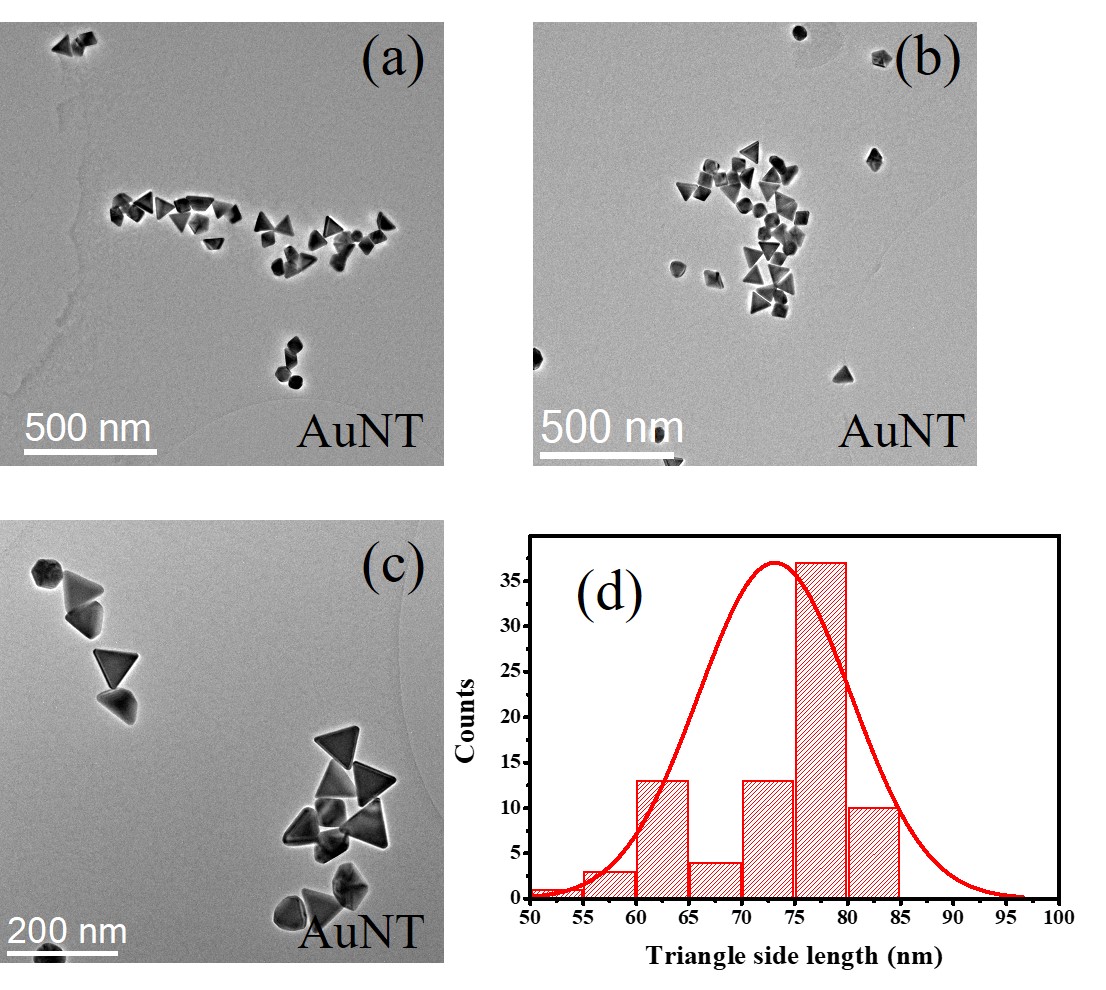


**Figure S1:** (a) – (c) BF-TEM images of the bare AuNT in different regions of the grid. (d) Histogram of the particle size (Au nanotriangle side length) analysis showing an average AuNT side length of 73.11 ± 7.21 nm.


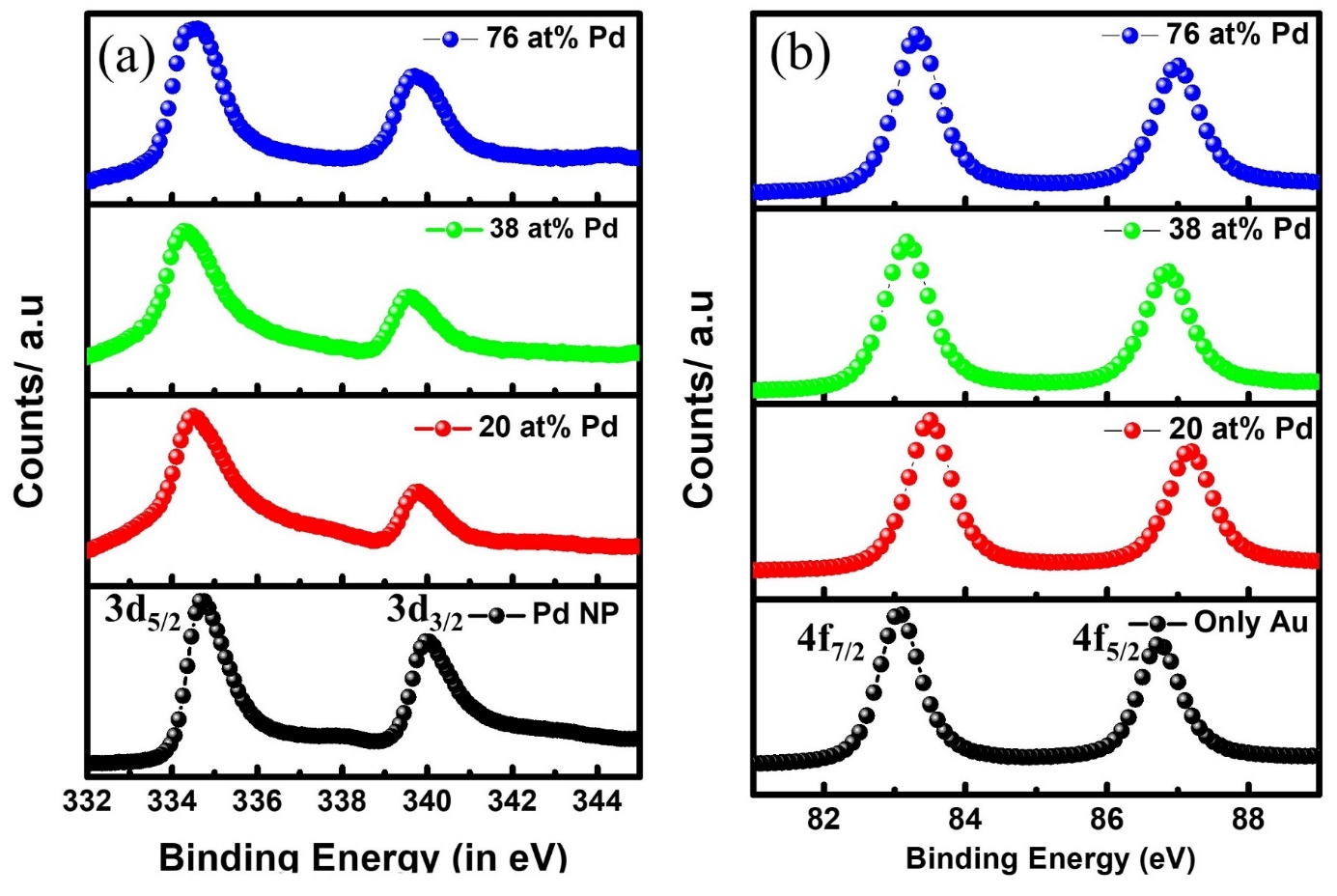


**Figure S2:** (a) High-resolution XPS spectra of the evolution of Pd3d_5/2_ and Pd_3/2_ peaks for different AuNT@Pd core@shell nanostructures and bare Pd nanoparticles (in black) (b) High-resolution XPS spectra of the evolution of Au4f_7/2_ and Au4f_5/2_ peaks for different AuNT@Pd core@shell nanostructures and bare AuNT nanoparticles (in black).


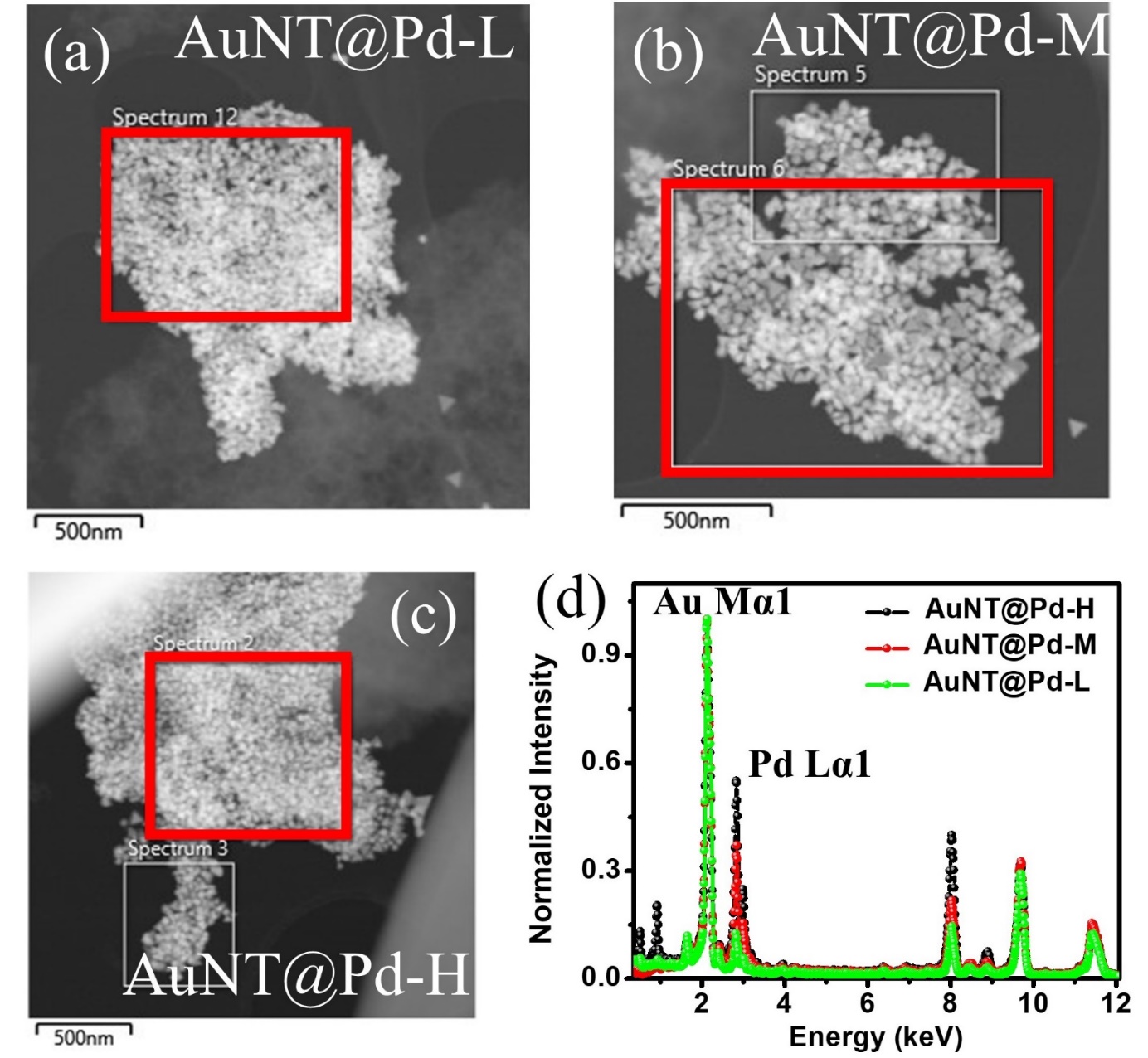


**Figure S3:** (a)-(c) STEM-HAADF images of AuNT@Pd-L, AuNT@Pd-M and AuNT@Pd-L showing different regions of EDS data acquisition. (d) Comparative EDS spectra for the three Pd doped samples normalized with respect to Au-M X-ray peak.


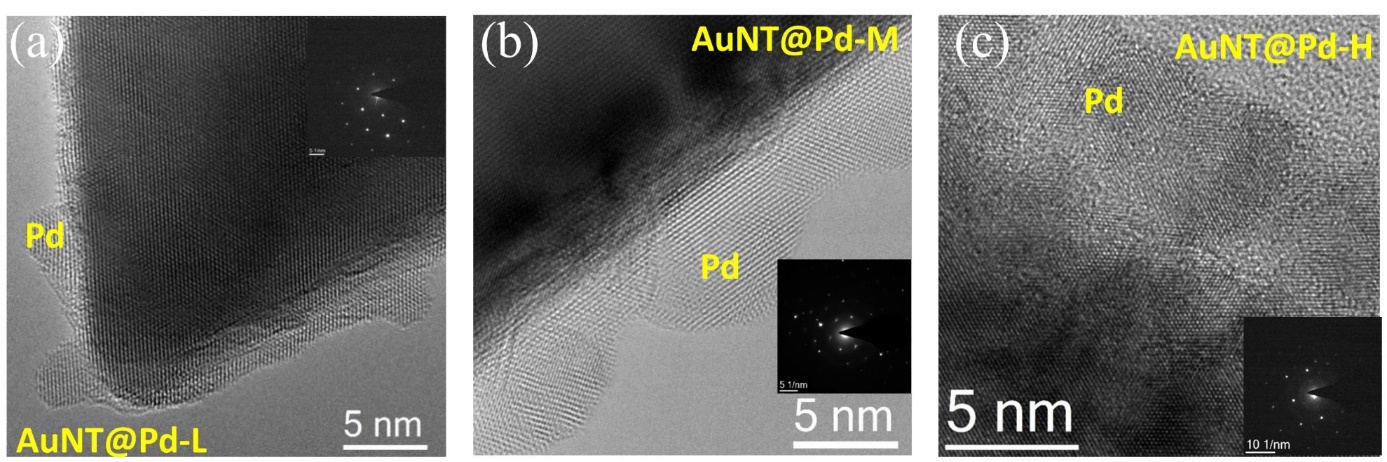


**Figure S4:** (a), (b) and (c) HRTEM images of NT with 13.9 at% Pd, 37.9 at% Pd and 48.3 at% Pd, respectively. Inset of (a), (b) and (c) show selected area electron diffraction (SAED) patterns taken from the nanoparticles.


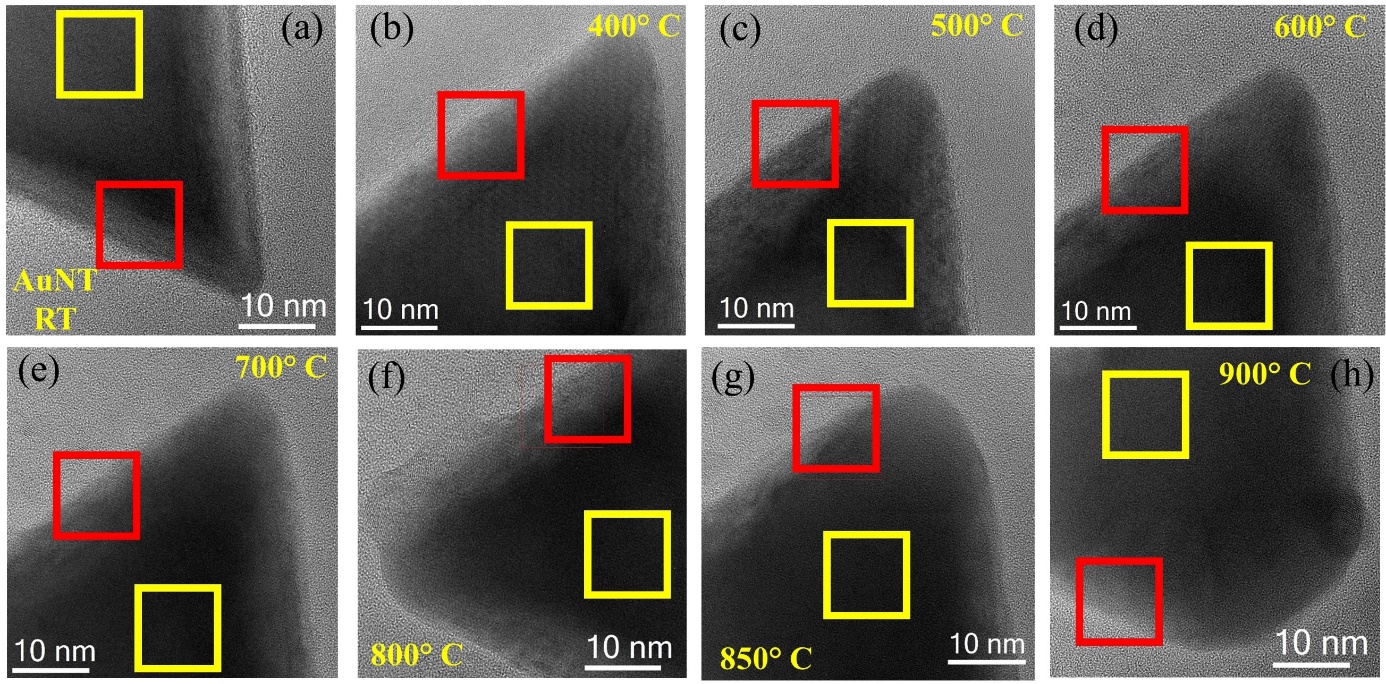


**Figure S5.** High-magnification images of AuNT at different temperatures: (a) RT, (b) 400°C, (c) 500°C, (d) 600°C, (e) 700°C, (f) 800°C, (g) 850°C, and (h) 900°C, respectively. FFT patterns (Figure S4) were obtained from the yellow and red square region from each image to study the variation of lattice parameter with temperature.


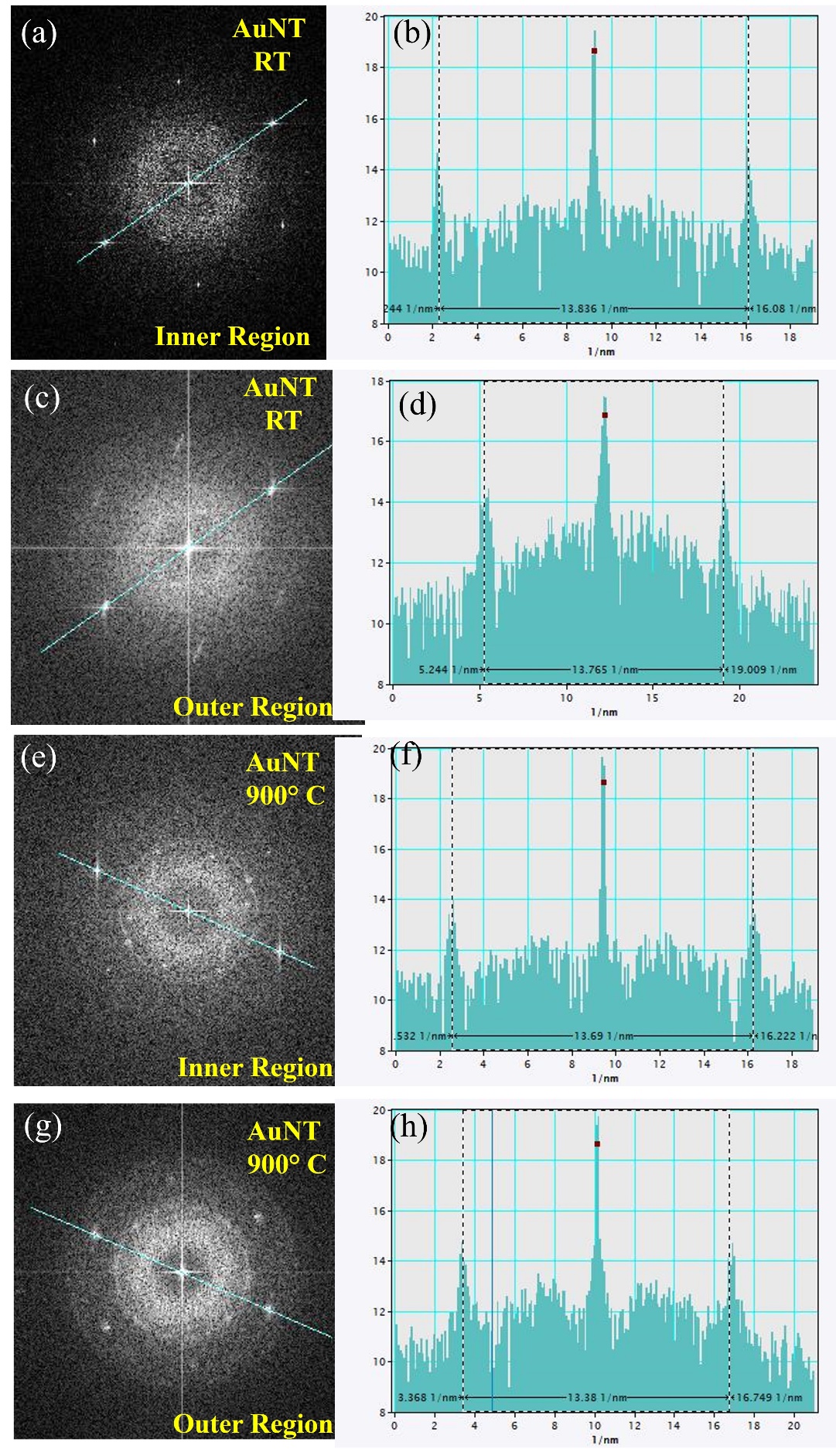


**Figure S6.** (a) FFT pattern obtained from the inner portion of AuNT at RT (marked by yellow square in Figure 8a. (b) Line profile obtained along the FFT spots to measure the lattice parameter. (c) and (d) are the same for the outer portion of AuNT at RT (marked by red square in Figure 8b. (e)-(g) FFT spots and line profile obtained along the FFT for AuNT heated at 900°C. The region is marked by yellow square (for inner region) and (red square) for outer region in Figure 8h.


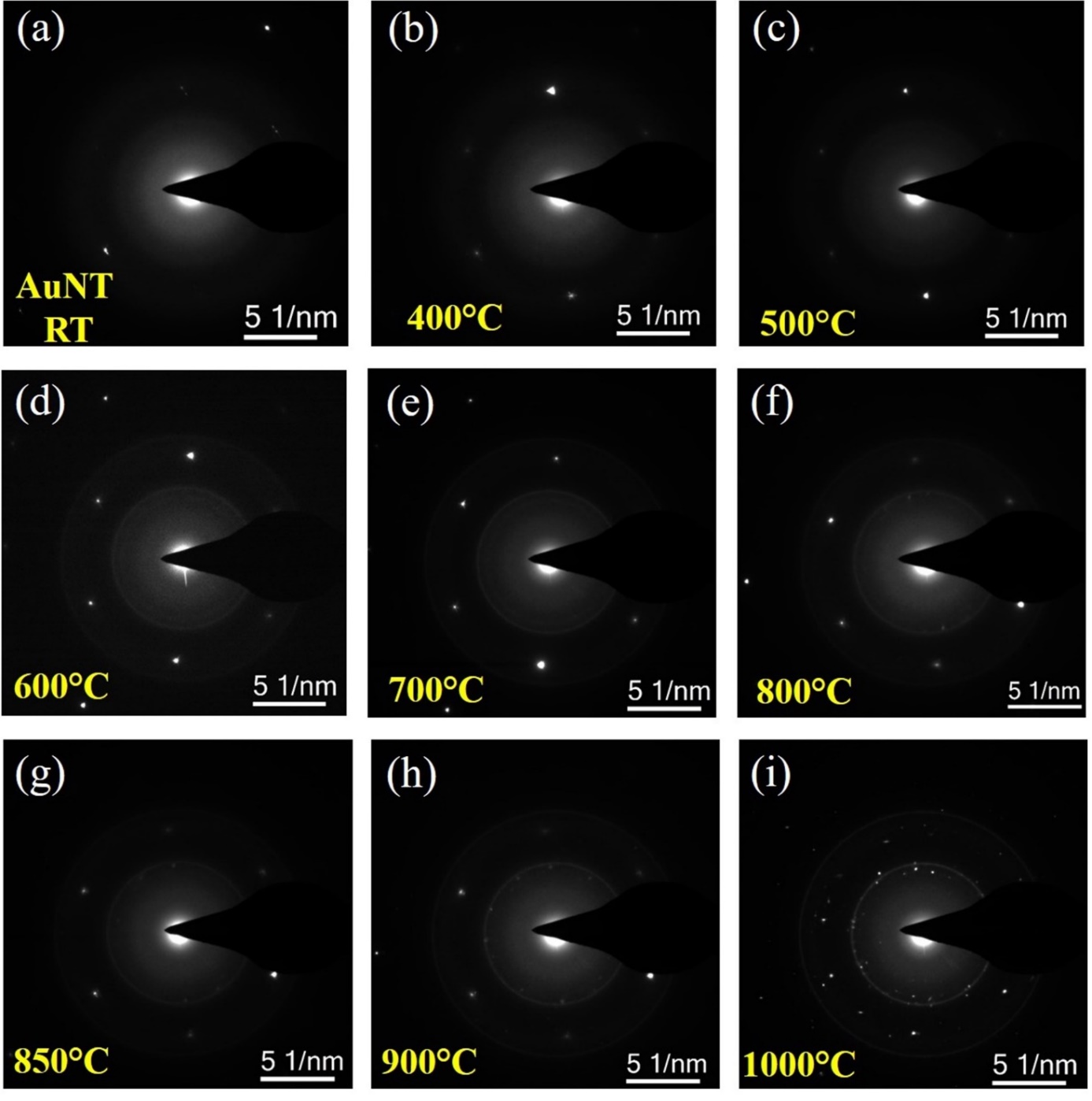


**Figure S7.** Variation of SAED patterns of AuNT at different heating temperatures. The generation of ring pattern at higher temperature shows formation of amorphous phases indicating a molten state of the nanoparticle.


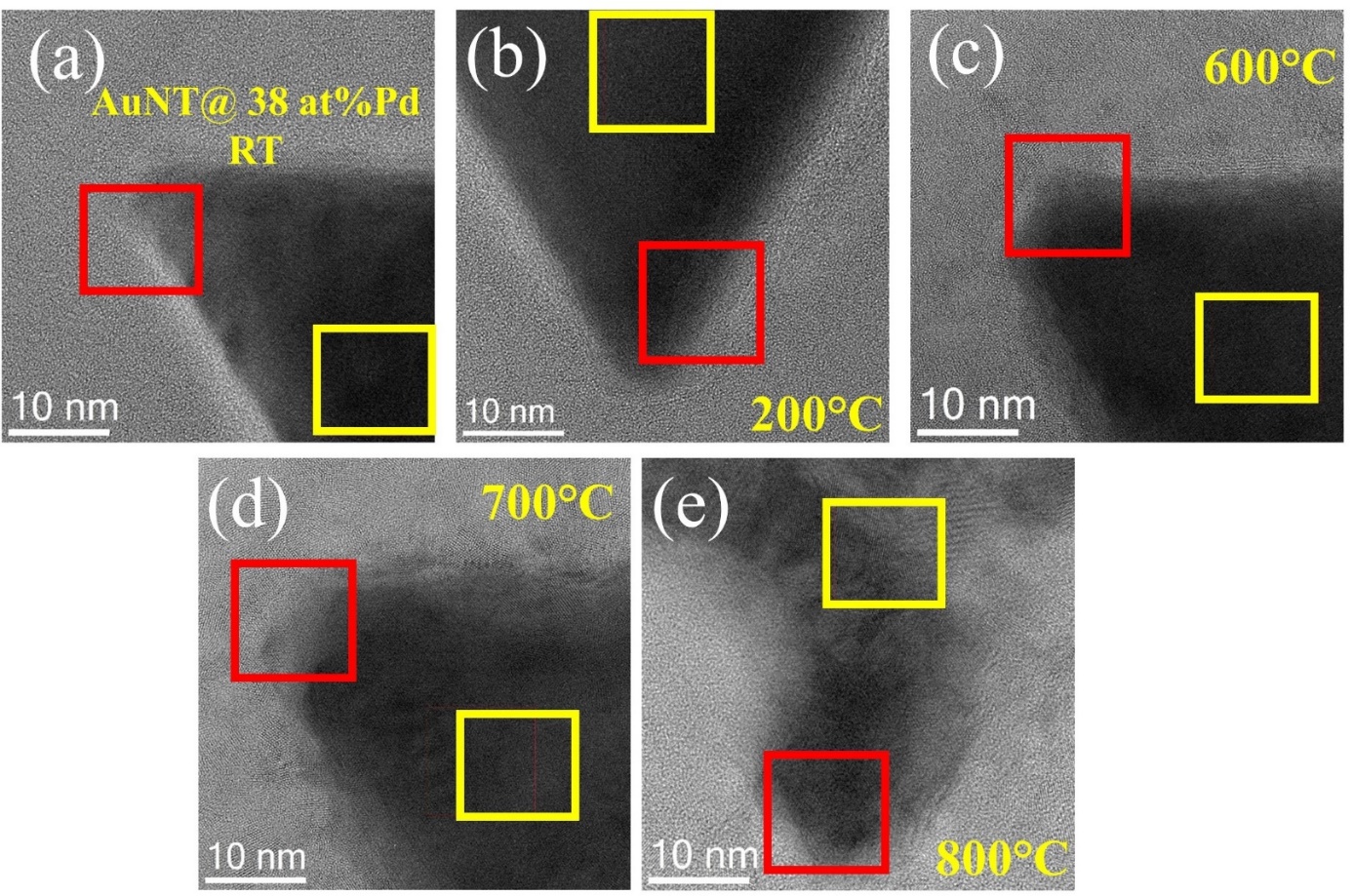


**Figure S8.** (a)-(e) HRTEM images of an AuNT@Pd-L NP with inner and outer regions of the NP marked by yellow and red squares, respectively, at different in-situ heating temperatures. The regions were selected to obtain the FFT patterns for determining the interplanar spacing variation with temperature.


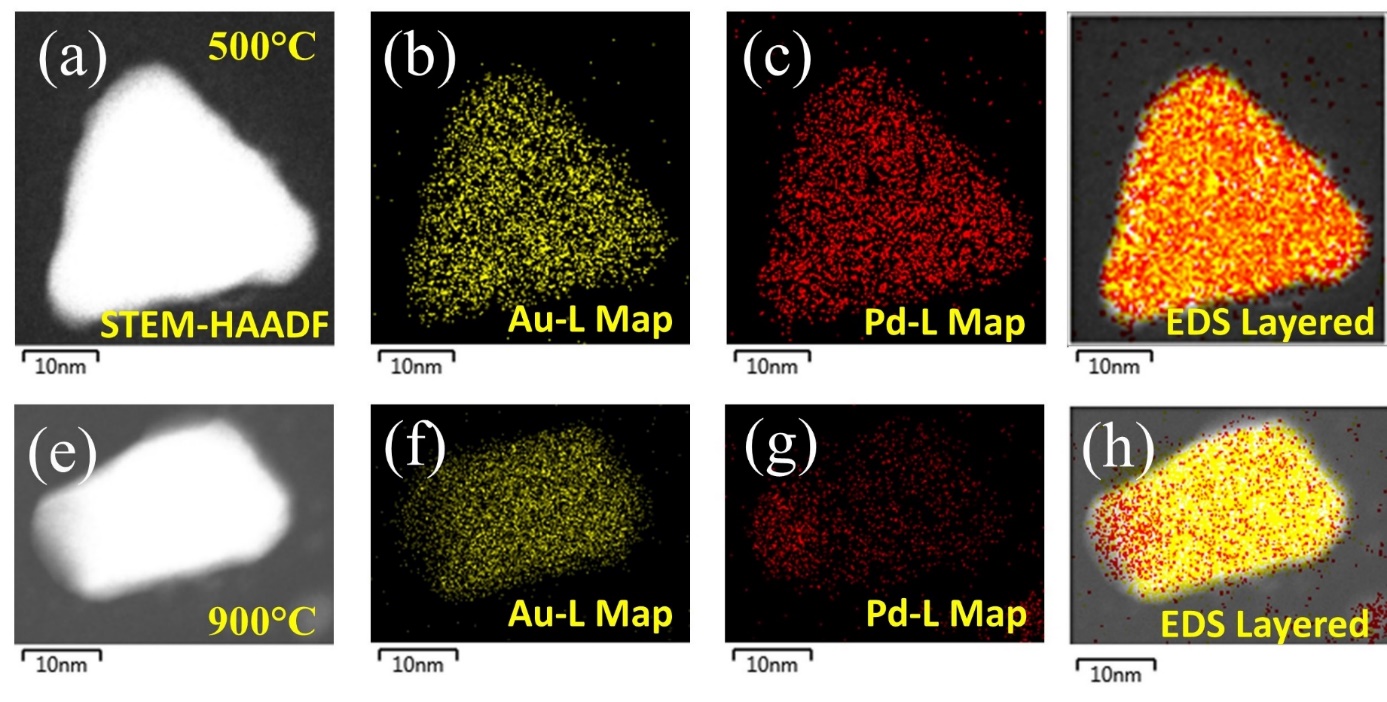


**Figure S9.** AuNT@Pd-M NP analysis. (a) STEM-HAADF image of Au-Pd alloy formed at 500°C. Figures (b), (c) and (d) show Au-L map, Pd-L map and EDS layered map showing uniform distribution of Au and Pd over the entire nanoparticle. (e) STEM-HAADF image of the same nanoparticle when annealed at 900°C. Figures (f)-(h) show Au-L map, Pd-L map and EDS layered image showing concentration or segregation of Pd at one side of the nanoparticle.


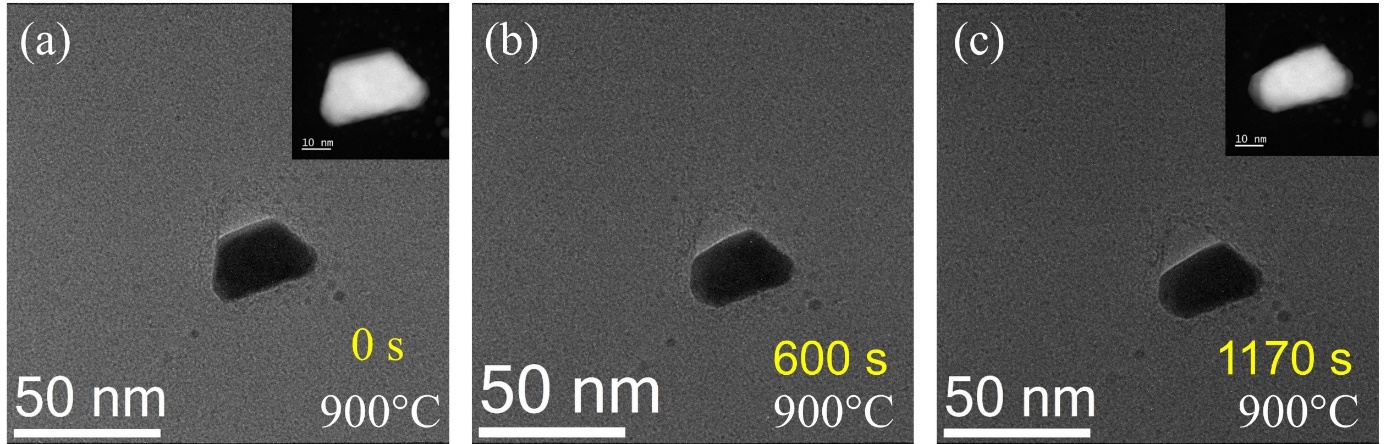


**Figure S10.** (a) BF-TEM image of AuNT@Pd-M at the start of heating the NP at 900°C. The inset shows the STEM-HAADF image where the whole nanoparticle is giving the same contrast indicating homogeneous mixing of Au and Pd. (b) TEM image of the NP at 600 s after the starting of heating showing deformation at the top left surface of the NP. (c) Image of the NP after 1170 s showing formation of Pd-rich region on the left side. The STEM-HAADF image shows bright and darker contrast, which corresponds to Au and Pd, respectively.


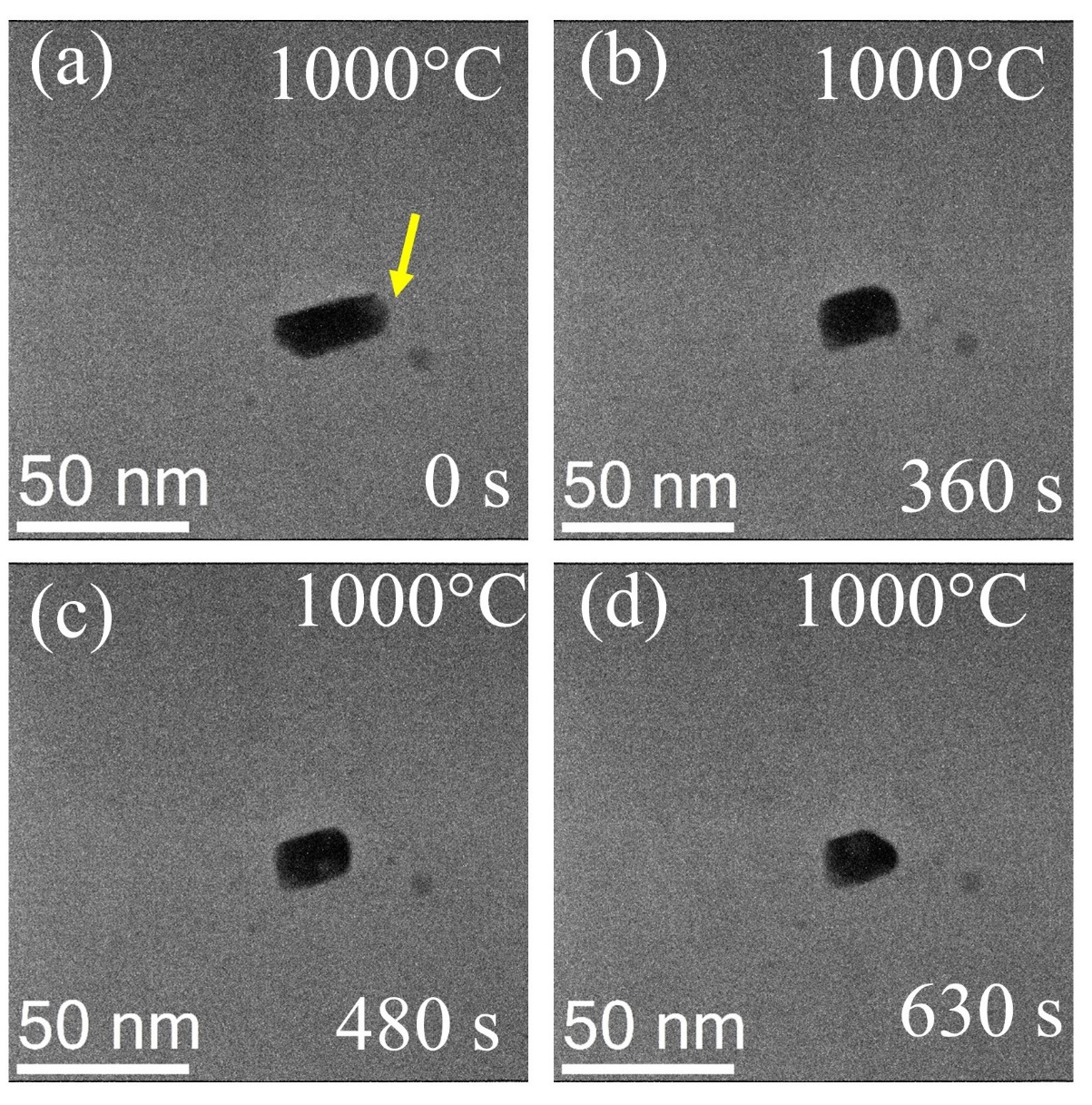


**Figure S11.** Decomposition of AuNT@Pd-M at 1000ºC at different time interval (a) 0 sec (b) 360 sec (c) 480 sec and (d) 630 sec.


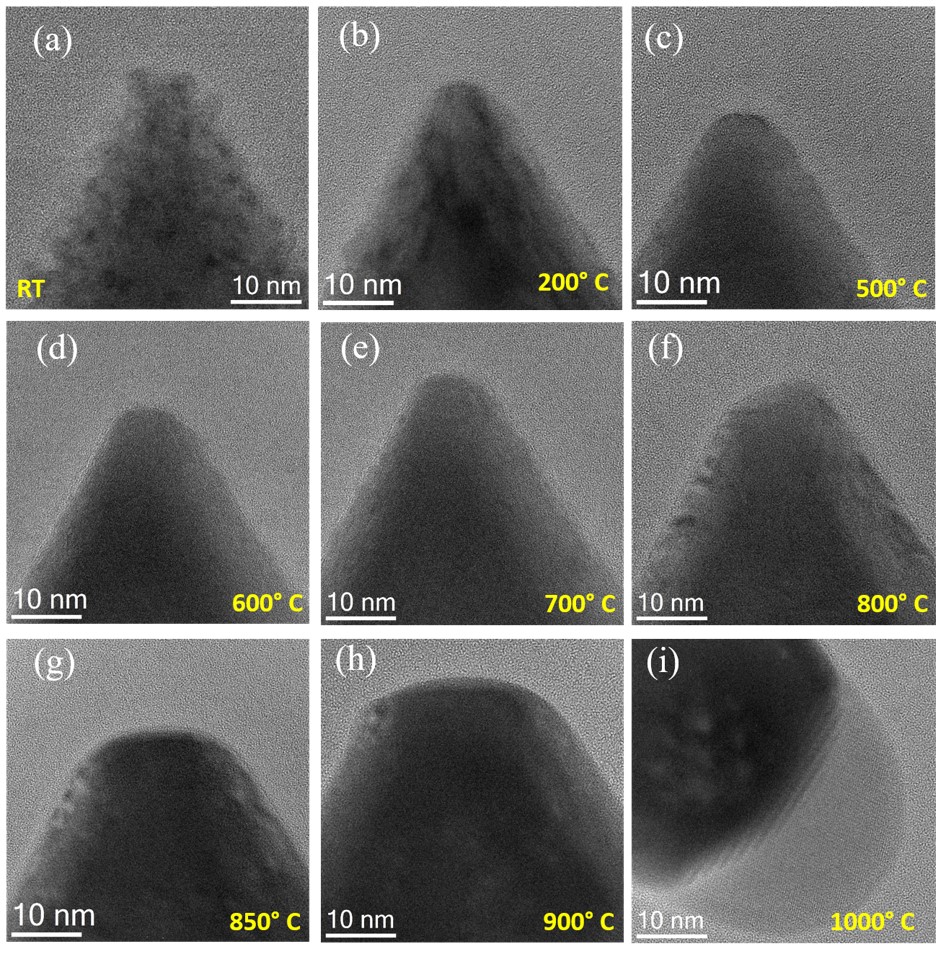


**Figure S12.** (a)-(i) HRTEM images of AuNT@Pd-H at different temperatures. This shows that initially, the Pd structure lost the columnar form at 200°C. Finally, the Au-Pd separation happens at 1000°C at a larger amount (Figure i) compared to the previous NP with lower Pd loading.


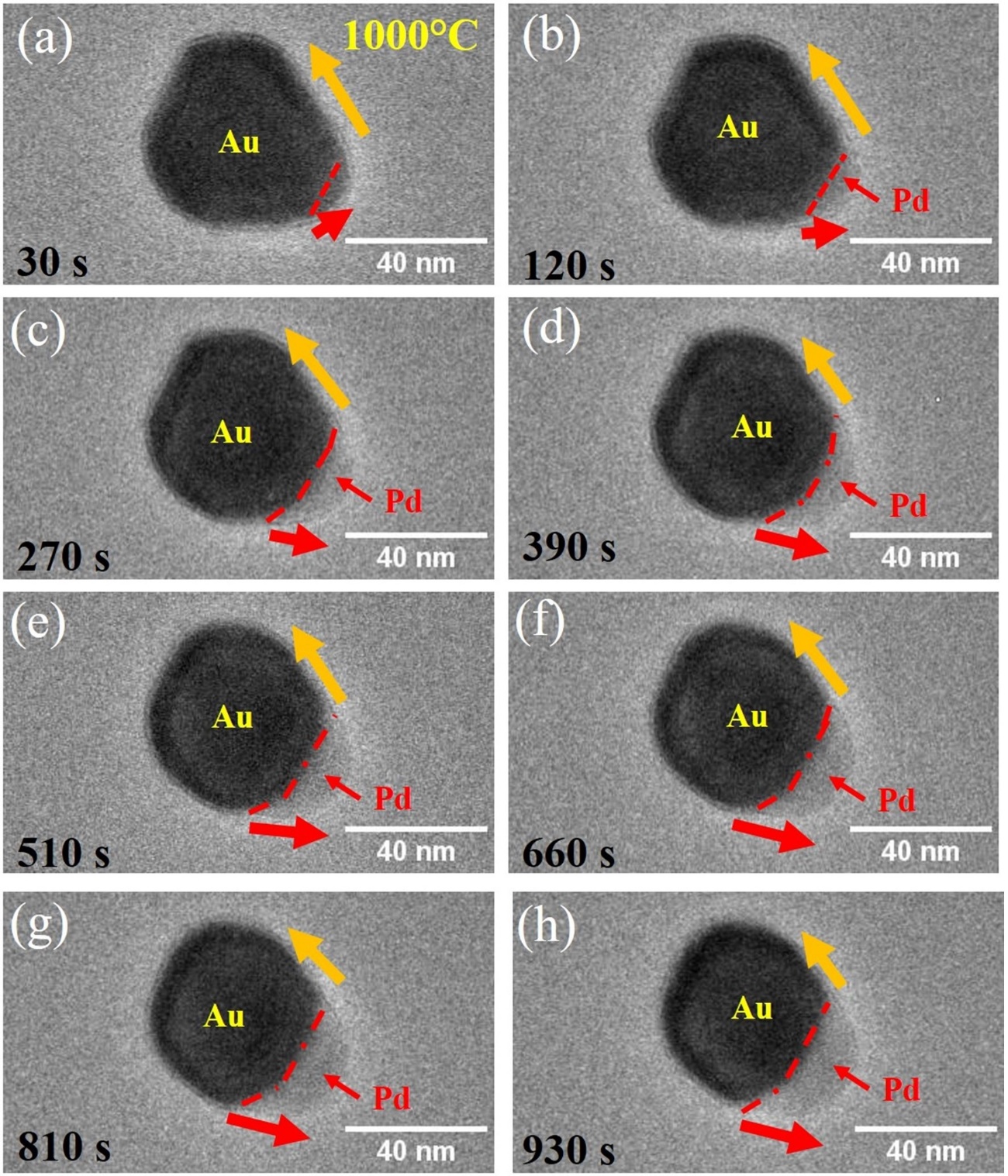


**Figure S13.** Formation process of the phase separated AuNT/Pd nanoparticle during heating at 1000°C at different times (a) 30 sec (b) 120 sec (c) 270 sec (d) 390 sec (e) 510 sec (f) 660 sec (g) 810 sec and (h) 930 seconds, respectively.


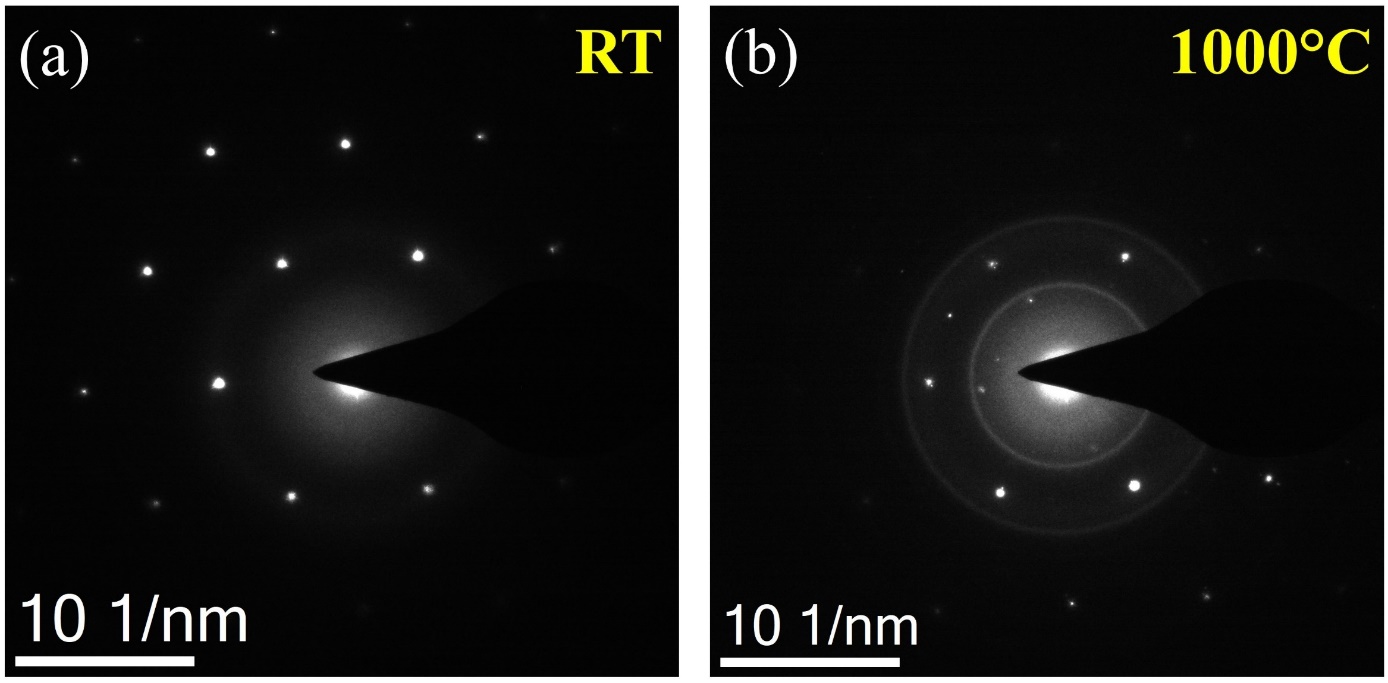


**Figure S14.** Evolution of SAED pattern of AuNT@Pd-H nanoparticle from (a) RT to (b) 1000°C, respectively.


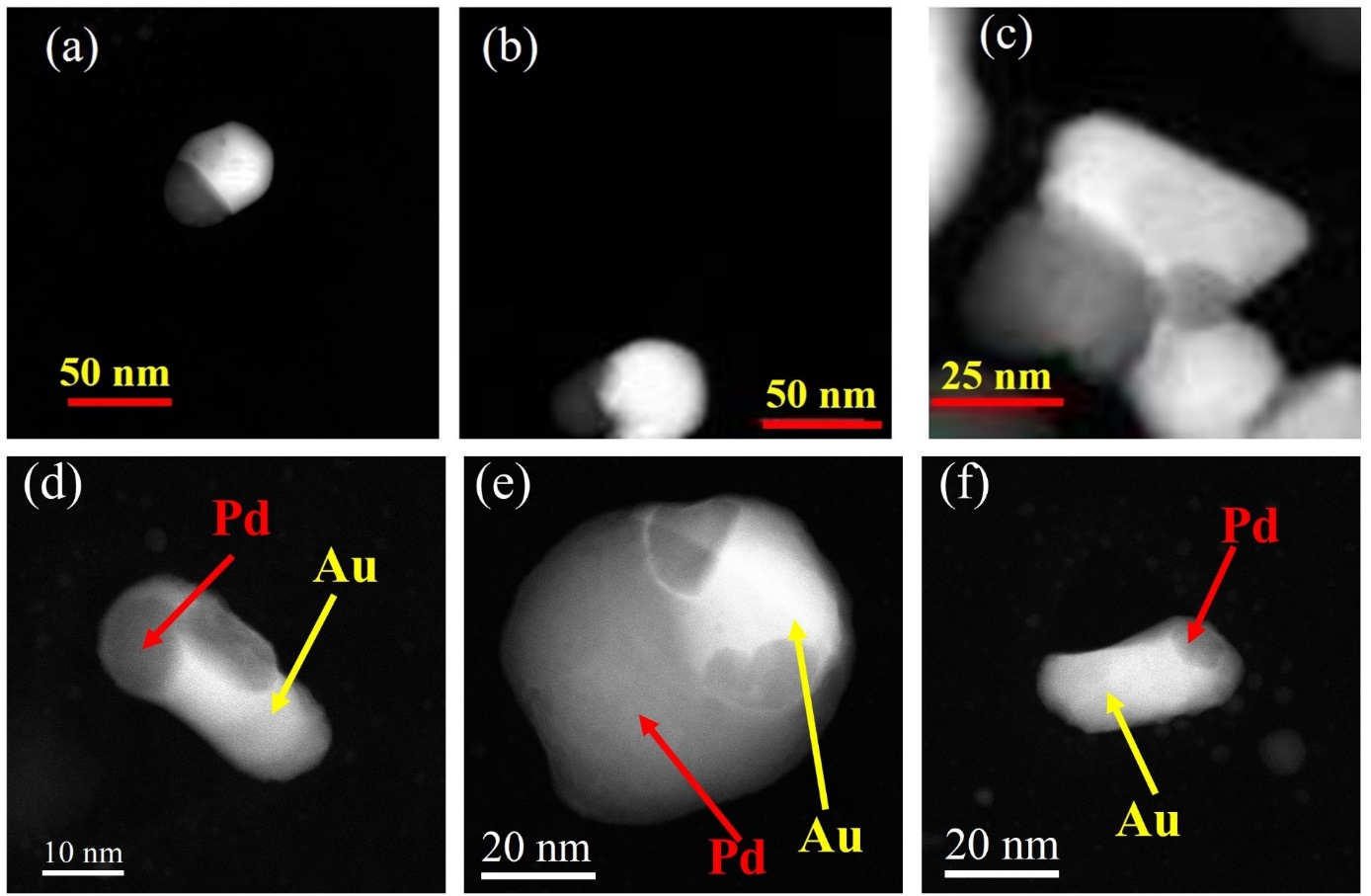


**Figure S15.** (a)-(f) STEM-HAADF images of different particles of AuNT@Pd-H sample, which showed Au-Pd separation at 1000°C and subsequent cooling at RT.


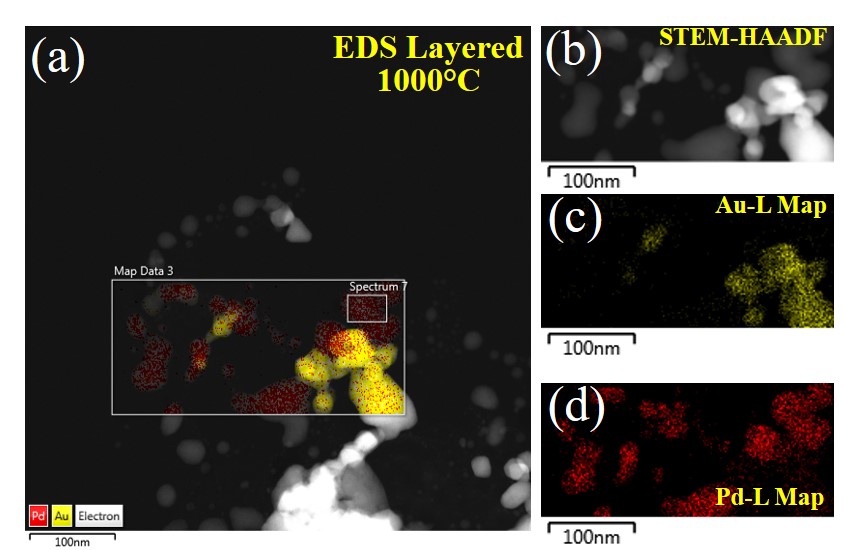


**Figure S16.** (a) STEM-HAADF and EDS layered images of phase separated Au/Pd clustered nanoparticle after heating to 1000°C and subsequent cooling at RT and keeping the sample in the ambient atmosphere for one week. (b)-(d) display an HAADF-STEM image, EDS (Au-L and Pd-L) maps showing distinct separation of Au and Pd upon annealing at 1000°C and subsequent cooling at RT.


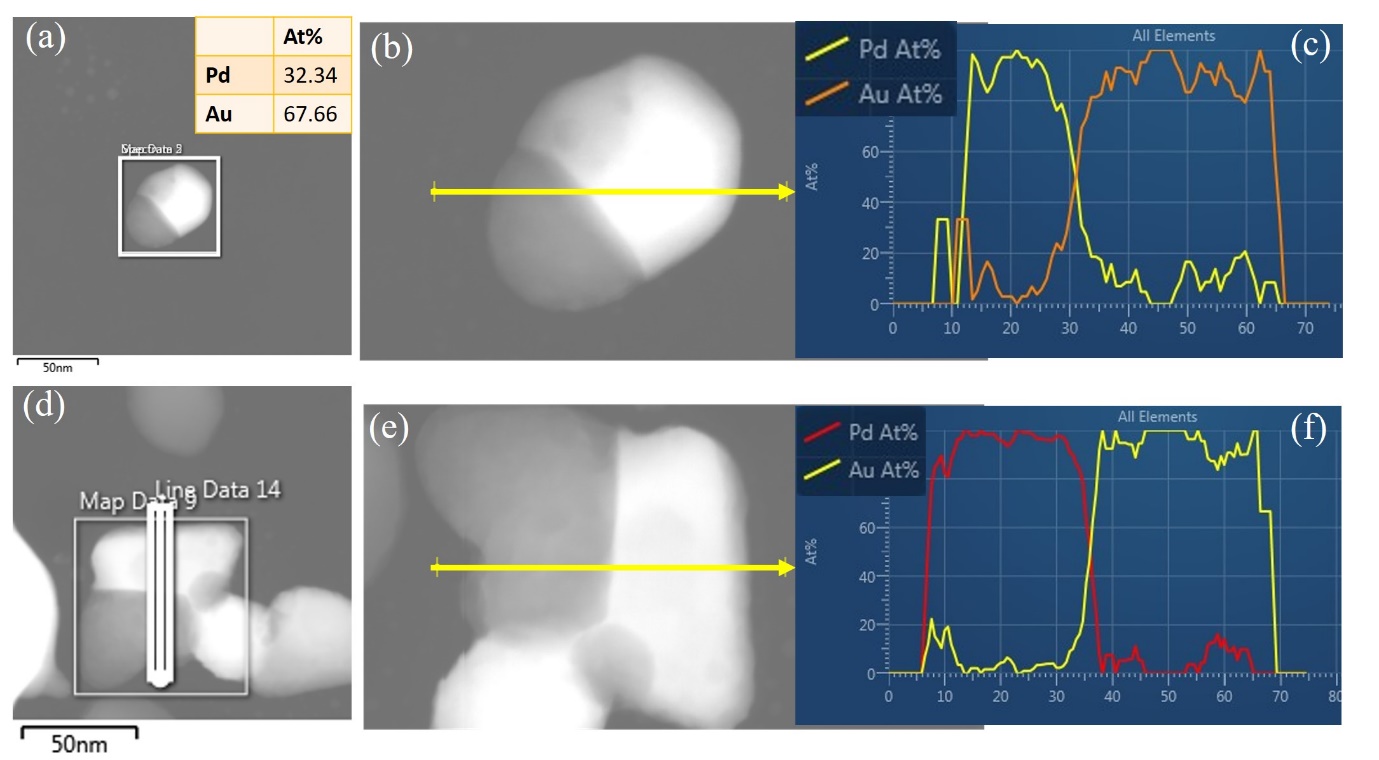


**Figure S17.** (a) and (c) STEM-HAADF images of phase separated Au-Pd NP after in-situ heating to 1000°C and subsequent cooling at RT and keeping the sample in the ambient atmosphere for one week. (c) and (d) EDS line profile taken along the Pd and Au-rich sides showing clear separation.


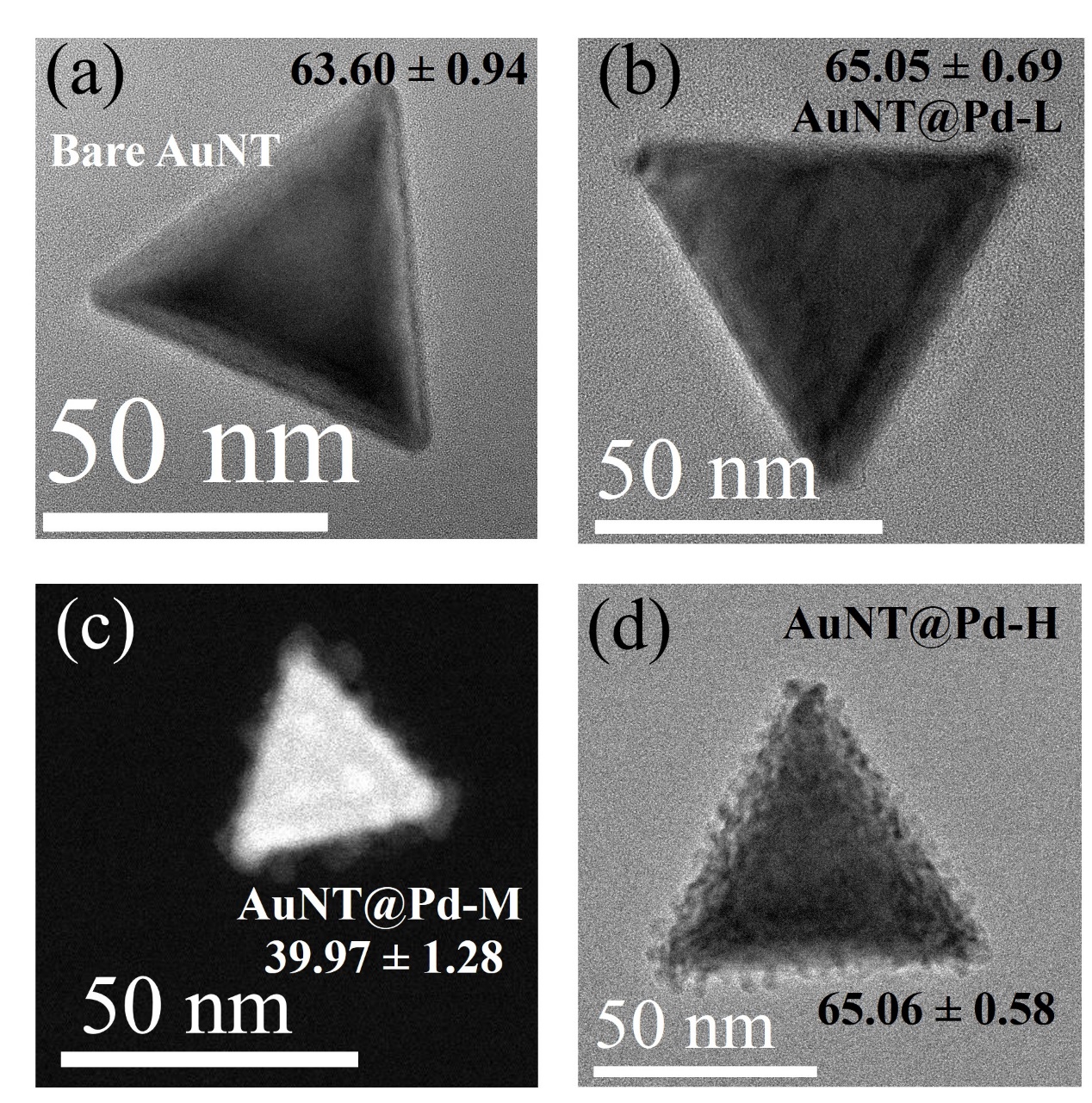


**Figure S18.** (a)-(d) BF-TEM and STEM-HAADF image of the different Pd doped AuNT nanoparticles with their average triangle side length, which were tracked during the in-situ heating process.

**Figure S19.** (a)-(i) STEM-HAADF image of the Janus nanostructures for the AuNT@Pd-H sample after keeping them in ambient atmosphere for one year.

**Figure S20.** (a)-(i) STEM-HAADF image of the janus nanostructured for the AuNT@Pd-H sample after keeping them in ambient atmosphere for one year.

**Figure S21.** (a), (e) and (f) Shows STEM-HAADF image, (b)-(c), (f)-(g), and (j)-(k) Shows EDS mapping and (d),(h) and (l) Shows EDS line profile of the janus nanostructured for the AuNT@Pd-H sample after keeping them in ambient atmosphere for one year.

**Detailed oxidation study using EDS**

Detailed EDS analysis of the Janus nanostructure (Figure S16, SI and Figure S17, SI), showed that the amount of O was very low on the nanostructure (3.58 at%, on the Au dominated region and 5.12 at at% on the Pd dominated region, Figure S17b and S17c), while higher amount of O was present on the background (10.86 at%, Figure S17d, and 15.81 at%, Figure S17e), indicating Au also play an important role to prevent Pd from oxidation even at high temperature. Detailed EDS analysis done on another particle shown in Figure S18, SI and in Figure S19, SI also showed similar results on the O distribution.


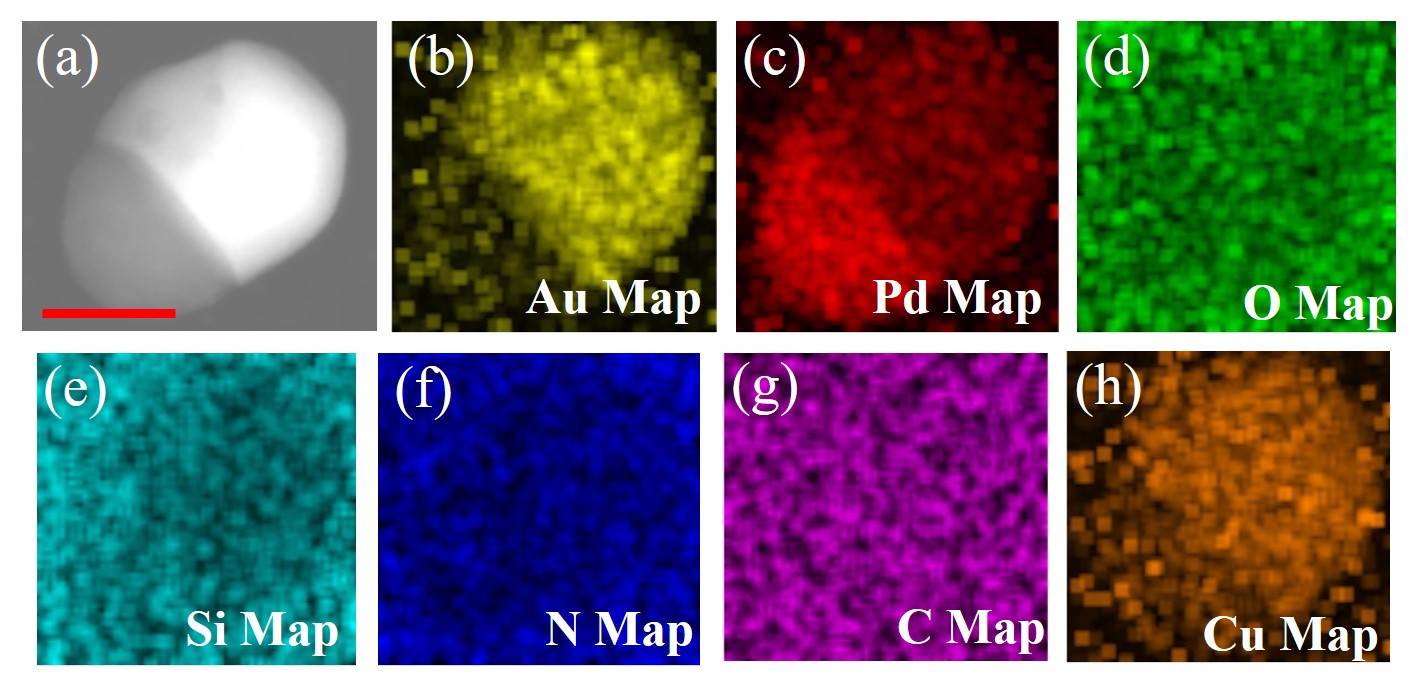


**Figure S22**. Detailed EDS quantitative maps of the Janus phase-separated nanostructure after heating at 1000°C and subsequent cooling to RT and keeping the sample in the ambient atmosphere for one week. (a) STEM-HAADF image. Figures (b)-(h) shows quantitative EDS map of Au, Pd, O, Si, N, C and Cu, respectively. Scale bar 25 nm. The O signal is depleted in the NT region clearly indicating the absence of oxidation in the nanostructure. Si, N and C stem from the used chip (carbon-coated silicon nitride), while Cu originates from fluorescence signal generated in the contacts. The Cu signal is increased in the nanostructure as fluorescence is increased for the high energy lines of Au and Pd.


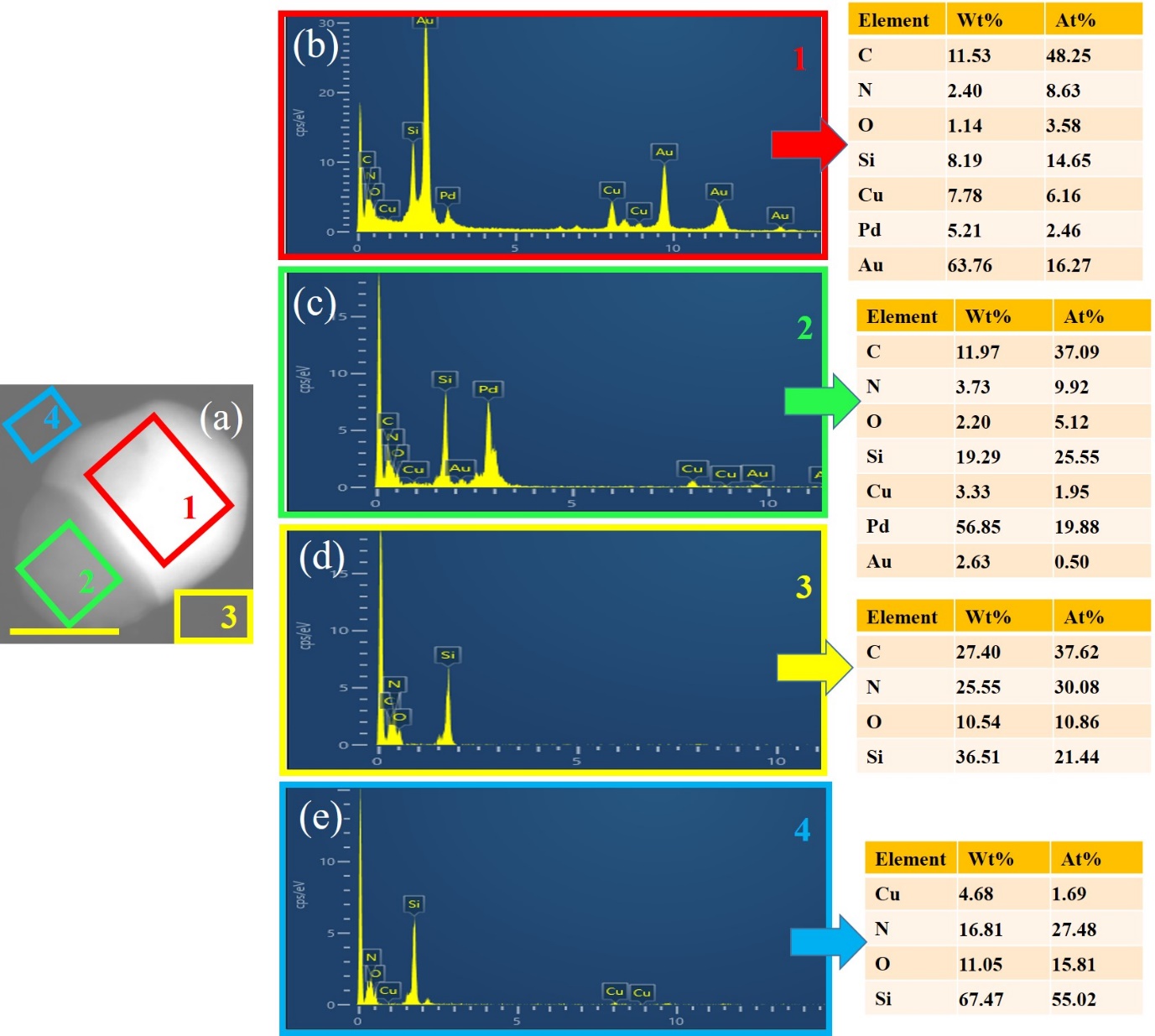


**Figure S23**. (a) STEM-HAADF image of the Janus phase-separated nanostructure after heating at 1000°C and subsequent cooling to RT and keeping the sample in the ambient atmosphere for one week. Figures (b)-(e) show acquired spectra from taken from different regions i.e. Au dominated region (Region 1), Pd dominated region (Region 2), and on the membrane of the in-situ heating chip (Region 3 and region 4), respectively. Oxygen content is increased in the membrane areas (also relative to Si and N) indicating the absence of oxidation of the nanostructure. Scale bar 25 nm.


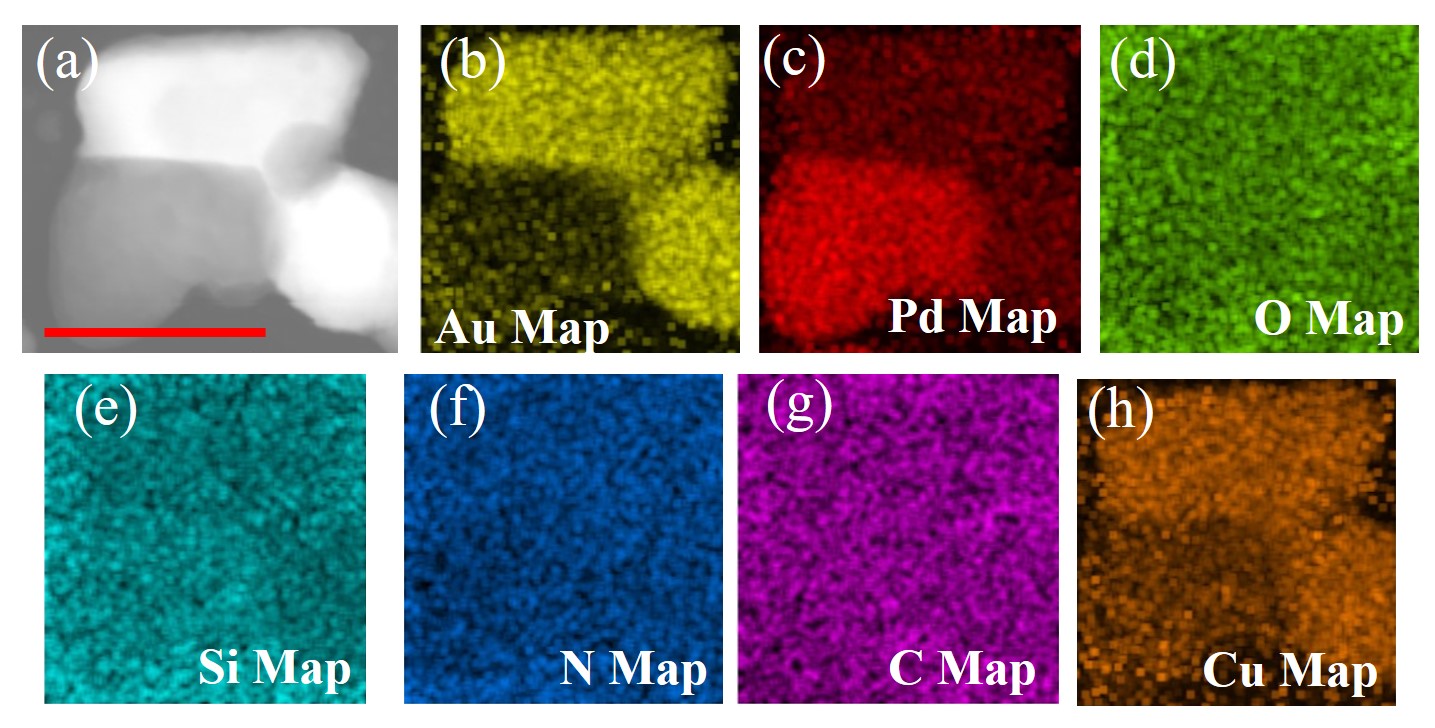


**Figure S24**. Detailed EDS quantitative maps of another phase-separated nanostructure after heating at 1000°C and subsequent cooling to RT and keeping the sample in the ambient atmosphere for one week. (a) STEM-HAADF image. Figures (b)-(h) shows quantitative EDS map of Au, Pd, O, Si, N, C and Cu, respectively. Scale bar 50 nm. The O signal is depleted in the NT region clearly indicating the absence of oxidation in the nanostructure. Si, N and C stem from the used chip (carbon-coated silicon nitride), while Cu originates from fluorescence signal generated in the contacts. The Cu signal is increased in the nanostructure as fluorescence is increased for the high energy lines of Au and Pd.


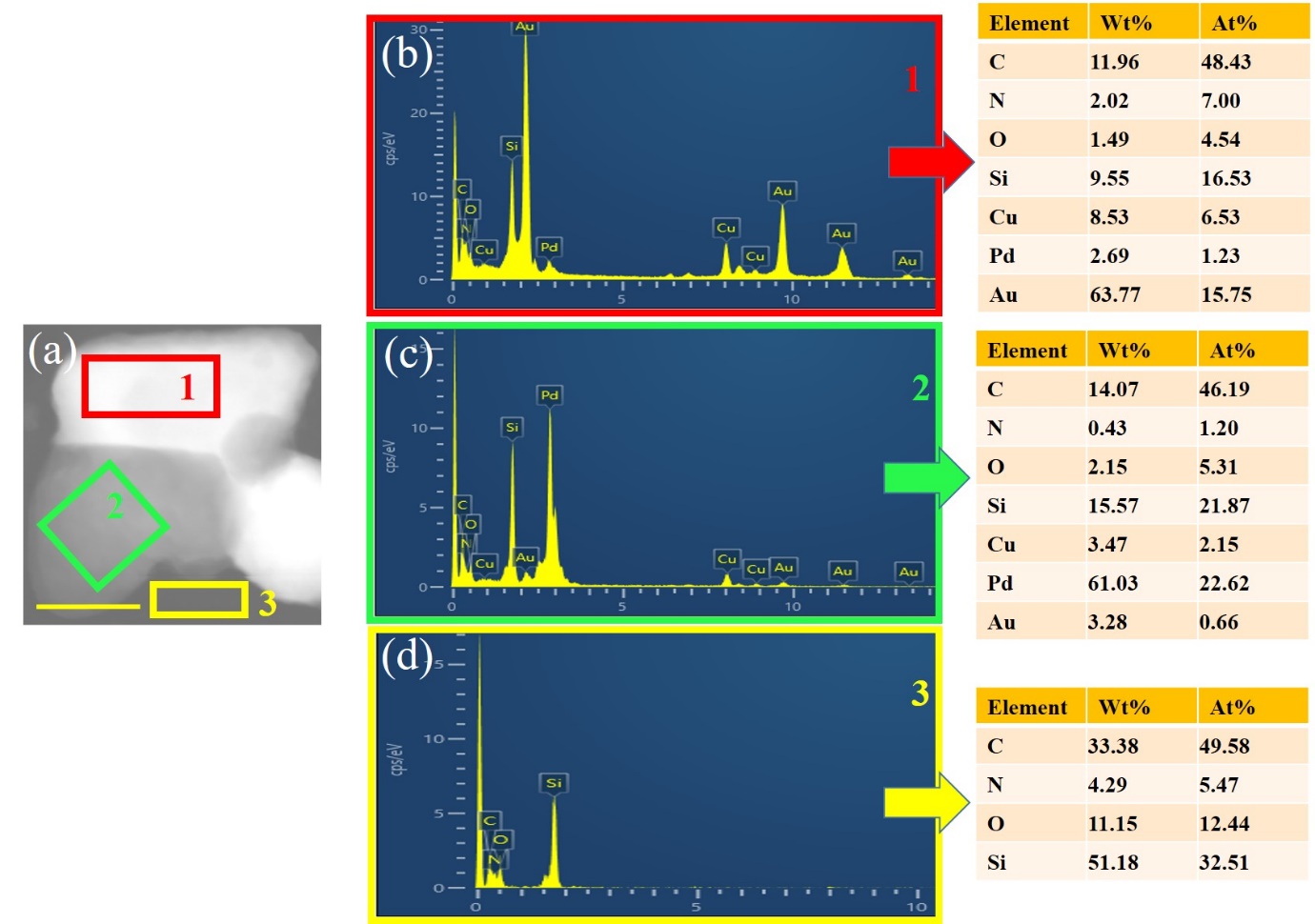


**Figure S25**. (a) STEM-HAADF image of another phase-separated nanostructure after heating at 1000°C and subsequent cooling to RT and keeping the sample in the ambient atmosphere for one week. Figures (b)-(d) show acquired spectra from taken from different regions i.e. Au dominated region (Region 1), Pd dominated region (Region 2), and on the membrane of the in-situ heating chip (Region 3), respectively. Oxygen content is increased in the membrane area (also relative to Si and N) indicating the absence of oxidation of the nanostructure. Scale bar 25 nm.


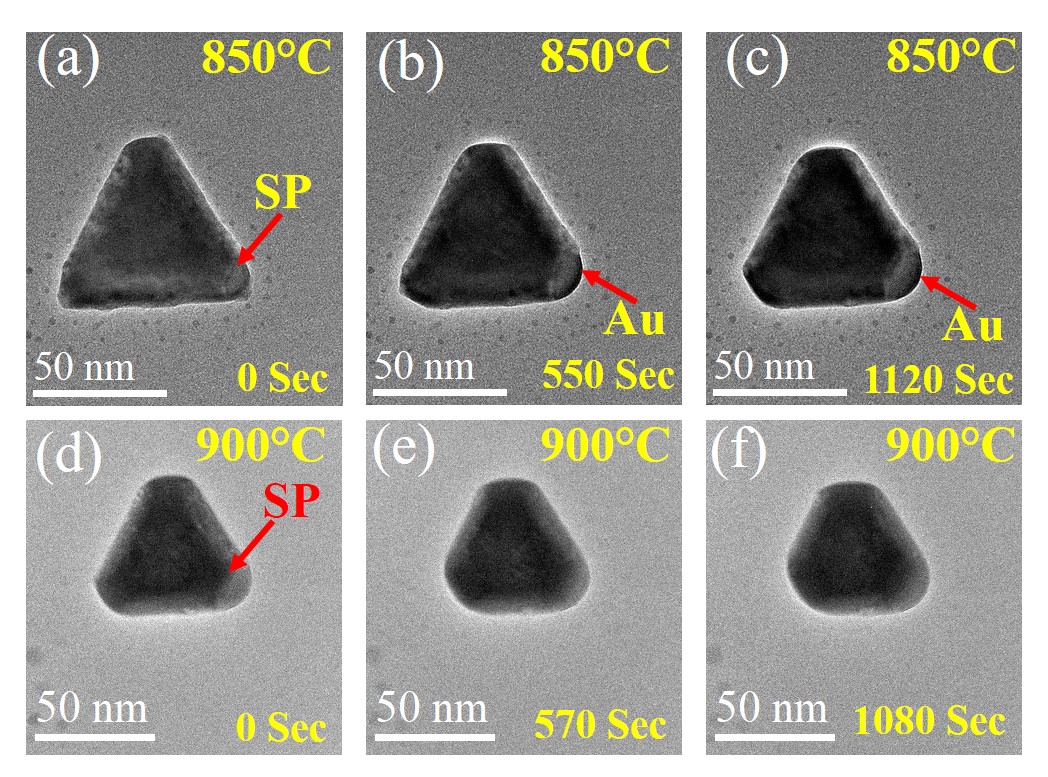


**Figure S26**. (a)-(c) BF-TEM images of variation of AuNT@Pd-H nanoparticle during heating at 850°C, showing initial surface pre-melting at the start and subsequent filling of the surface site by Au atoms (indicated by red arrow in Figure (b) and (c). (d)-(e) BF-TEM images of variation of AuNT@Pd-H nanoparticle during heating at 900°C, showing again initial surface pre-melting at the start and subsequent formation of Au-Pd alloy at the surface.
